# Treatment of unacceptable bleeding in long-term users of 52-mg levonorgestrel intrauterine device: a prospective observational study

**DOI:** 10.1016/j.xagr.2025.100474

**Published:** 2025-03-20

**Authors:** Patty A.H.H. van der Heijden, Karlijn A. Röttgering, Tamara J. Oderkerk, Jeanne P. Dieleman, Arianne C. Lim, Marlies Y. Bongers, Peggy M.A.J. Geomini

**Affiliations:** a Anna Hospital, Geldrop, The Netherlands (Heijden); b Grow - Research School of Oncology and Reproduction, Maastricht University, Maastricht, The Netherlands (Heijden, Lim, and Bongers); c Maxima Medical Center Veldhoven, Veldhoven, The Netherlands (Röttgering, Oderkerk, Dieleman, Bongers, and Geomini); d Maastricht University, Maastricht, The Netherlands (Lim)

**Keywords:** Estradiol, irregular bleeding, levonorgestrel intrauterine device, satisfaction, treatment

## Abstract

**BACKGROUND:**

In the first month after the insertion of the 52-mg levonorgestrel intrauterine device, irregular vaginal bleeding often occurs. In 6% to 18% of 52-mg levonorgestrel intrauterine device users, irregular vaginal bleeding reoccurs or continues for more than 6 months after insertion of the device. This study hypothesized that the addition of estradiol may be beneficial for the regeneration of the endometrium and may consequently decrease irregular bleeding, as these basal vessels might be shielded by the regenerated endometrium.

**OBJECTIVE:**

This prospective observational study aimed to evaluate the effectiveness of estradiol in treating irregular bleeding in patients who have had a 52-mg levonorgestrel intrauterine device in place for at least 6 months.

**STUDY DESIGN:**

The study was conducted in 2 hospitals and in 1 general practice in the Netherlands. Patients with a 52-mg levonorgestrel intrauterine device who experienced irregular bleeding and who chose to undergo estradiol treatment were included. Treatment consisted of 2 mg of oral estradiol daily for 6 weeks. The primary outcome was the number of bleeding days a month after 3 months of estradiol treatment compared with baseline. The secondary outcomes included the number of bleeding days 12 months after the start of medication, bleeding patterns, discontinuation rate of the 52-mg levonorgestrel intrauterine device, side effects, adverse events, and patient satisfaction at 3 and 12 months of follow-up.

**RESULTS:**

A total of 39 patients provided informed consent and completed the baseline questionnaires. The mean number of bleeding days decreased significantly from 22.5 days per month at baseline to 12.8 days per month 3 months after starting estradiol treatment. The 52-mg levonorgestrel intrauterine device was removed in 10.3% of patients at 3 months of follow-up and 33.3% of patients at 12 months of follow-up. The number of women reporting acceptable bleeding patterns and satisfaction with the 52-mg levonorgestrel intrauterine device increased substantially over 12 months of follow-up.

**CONCLUSION:**

A decrease in the number of bleeding days was observed in long-term 52-mg levonorgestrel intrauterine device users who experienced unfavorable bleeding after the administration of estradiol for 6 weeks. In addition, satisfaction rates increased significantly.

**Clinical trial registration number:**

NL8007

**AJOG Global Reports at a Glance**

**Why was this study conducted?**

Women with 52 mg levonorgestrel intrauterine devices (LNG-IUDs) in place may experience less irregular bleeding after six months of use if estradiol is added to promote endometrial regeneration.

**Key findings**

The mean number of bleeding days decreased significantly from 22.5 days per month at baseline to 12.8 days per month 3 months after instigation of estradiol treatment.

**What does this add to what is known?**

This is important to take into account when counselling patients about their options in case of bleeding complaints during long-term 52 mg LNG-IUD use.

## Introduction

An intrauterine device (IUD) is one of the most common tools used for contraception. Worldwide, approximately 17% of women use an IUD.^1^ The levonorgestrel-releasing IUD (52-mg LNG-IUD, 52 mg levonorgestrel [Mirena]) can be used as a treatment for heavy menstrual bleeding (HMB).^2^ To date, it can be used for both contraception and HMB for up to 8 years.^1^ The 52-mg LNG-IUD releases levonorgestrel into the uterine cavity. Levonorgestrel is a hormone that induces atrophy of the endometrial tissue. In the first month after insertion of the 52-mg LNG-IUD, irregular vaginal bleeding often occurs. After the first month, most women have no vaginal bleeding or little vaginal bleeding^3^ (see also Maldonado 2022). However, in 6% to 18% of 52-mg LNG-IUD users, irregular vaginal bleeding reoccurs or continues for more than 6 months after insertion of the device.^4^^,^^5^ Hypothetically, irregular vaginal bleeding might occur because the levonorgestrel released by the 52-mg LNG-IUD can lead to a downregulation of estrogen receptors in the stroma cells, which results in diminished endometrial proliferation. Consequently, endometrial atrophy occurs, and basal blood vessels in the endometrium become vulnerable. Because of the combination of the atrophic endometrium and an increase of vulnerable blood vessels in the mucosa, irregular vaginal bleeding can occur.^6^, ^7^, ^8^ The Dutch guideline for general practitioners recommends treatment with daily oral estradiol for 52-mg LNG-IUD users (short or long term) with irregular bleeding patterns (https://www.nhg.org/standaarden/volledig/nhg-standaard-vaginaal-bloedverlies). However, evidence supporting this treatment is lacking.

We hypothesized that the addition of estradiol may be beneficial for the regeneration of the endometrium and may consequently decrease irregular bleeding, as these basal vessels might be shielded by the regenerated endometrium. Women with a 52-mg LNG-IUD in place may experience less irregular bleeding after 6 months of use if estradiol is added to promote endometrial regeneration.

This prospective observational study investigated whether the daily use of 2 mg of estradiol for 6 weeks reduces irregular bleeding in patients who have had a 52-mg LNG-IUD in place for at least 6 months.

## Materials and methods

### The research population

This prospective observational study was conducted between May 2017 and July 2022 in the Netherlands. Patients with a 52-mg LNG-IUD (Mirena) in place for at least 6 months who had irregular bleeding and chose to undergo estradiol treatment, which was the national protocol, were included by general practitioners in 1 general practice (GP) office and by gynecologists and residents in 2 teaching hospitals (the Maxima Medical Center in Veldhoven, The Netherlands, and the Maastricht University in Maastricht, The Netherlands). The patients were selected after an LNG-IUD had been in situ for >6 months. The insertions were performed by a general practitioner, midwife, or gynecologist at the office. Data on 52-mg LNG-IUD insertions were not collected. The study was conducted in a hospital setting where patients are referred, for example, by a general practitioner. Patients who were referred due to persistent bleeding while using a 52-mg LNG-IUD (placed 6 months earlier for any indication) were invited to participate in the study. We have no information on patients who were not referred to our clinic.

Irregular bleeding was defined as prolonged irregular bleeding or prolonged spotting complaints more than 6 months after insertion of a 52-mg LNG-IUD or undesirable vaginal bleeding after a period of amenorrhea >6 months after device insertion. The exclusion criteria were the presence of intrauterine fibroids and/or polyps, uterine malignancy, and/or an abnormal Papanicolaou test. In addition, patients with a contraindication for estradiol and patients aged <18 years were excluded from the study. The Medical Ethics Committee (METC) declared that the study did not fall under the Medical Research Involving Human Subjects Act (WMO) on May 9, 2017 (METC N17.070). Written informed consent was obtained from all patients.

After a pilot study (with a follow-up of 3 months) with the inclusion of 20 patients, data analysis was performed, and the results were published.^9^ We decided to continue the study and to expand the population size to 100 patients and to assess the outcomes at 12 months of follow-up to obtain more robust data.^10^ As an extended pilot study, no formal sample size calculation was performed. A protocol amendment to this effect was accepted by the concerned METC under the initial declaration on April 8, 2020 (METC 2020-1452).

### Baseline variables

The patient characteristics used to describe the study population included age, body mass index (BMI), endometrial thickness, smoking status, indication for insertion of the 52-mg LNG-IUD, number of previous 52-mg LNG-IUDs, and any treatments previously used for the current complaints. In addition, number of bleeding days, bleeding pattern, and satisfaction with the bleeding pattern were collected at baseline using questionnaires after hospital or GP visit and completion of the informed consent process.

### Methods, techniques, and statistics

Treatment consisted of 2 mg of oral estradiol daily for 6 weeks. If bleeding was persistent, no other treatment was offered. The primary outcome of the study was the mean number of bleeding days at the third month after treatment compared with the baseline. The secondary outcome measurements of the study were the mean number of bleeding days at the 12th month (after the start of estradiol treatment) compared with the baseline, the bleeding pattern, the discontinuation rate of the 52-mg LNG-IUD, the side effects and adverse events, and the satisfaction with the bleeding pattern at 3 and 12 months after the start of estradiol treatment. The questionnaires were collected at baseline; at 3 months after the start of treatment, including a menstrual bleeding chart; and at 12 months after the start of treatment. “No menstruation,” “normal menstruation,” and “occasional small amount of blood loss (spotting),” were menstruation patterns classified as acceptable bleeding pattern. “HMB,” “regular or irregular menstrual bleeding,” “blood loss of more than 14 days,” “postcoital bleeding,’’ and “continuous blood loss” were menstrual patterns classified as unacceptable bleeding patterns. Satisfaction was scored using a 10-point numeric rating scale ranging from 1 (unsatisfied) to 10 (satisfied). To calculate the satisfaction rates, satisfaction was dichotomized into unsatisfied (scores 1–4) and satisfied (scores 5–10).

This analysis included data from questionnaires and menstrual bleeding charts. Data were collected using anonymized written questionnaires and/or questionnaires completed by telephone. Data were analyzed using the SPSS (version 28; SPSS Inc). Categorical variables were expressed as numbers and percentages. Continuous data with a normal distribution were expressed as mean with SD. Continuous data with a non-normal distribution were expressed as median with IQR. The distributions were checked using histograms and skewness and kurtosis parameters. The Fisher exact test and chi-square test were used to analyze categorical data, as appropriate. To compare the change in means from baseline, the *t* test for paired samples was used, and 95% CIs were calculated as a measure of precision. Changes from baseline in categorical variables were analyzed using the McNemar test for paired samples. Questionnaire data on bleeding days, adverse events, and satisfaction scores completed while the 52-mg LNG-IUD was no longer in situ were excluded from the analyses. The time to removal of the 52-mg LNG-IUD from the start of estradiol treatment was analyzed using Kaplan-Meier survival analysis. Data lacking from returned questionnaires were registered as missing. If data on 52-mg LNG-IUD removal were missing at 12 months while the 52-mg LNG-IUD was known to be in place at 3 months, they were imputed as having been removed at 90 days (ie, after 3 months of follow-up). Otherwise, missing data were not imputed. Statistical significance was accepted at a *P* value of <.05.

## Results

### Baseline characteristics

**Figure 1:**
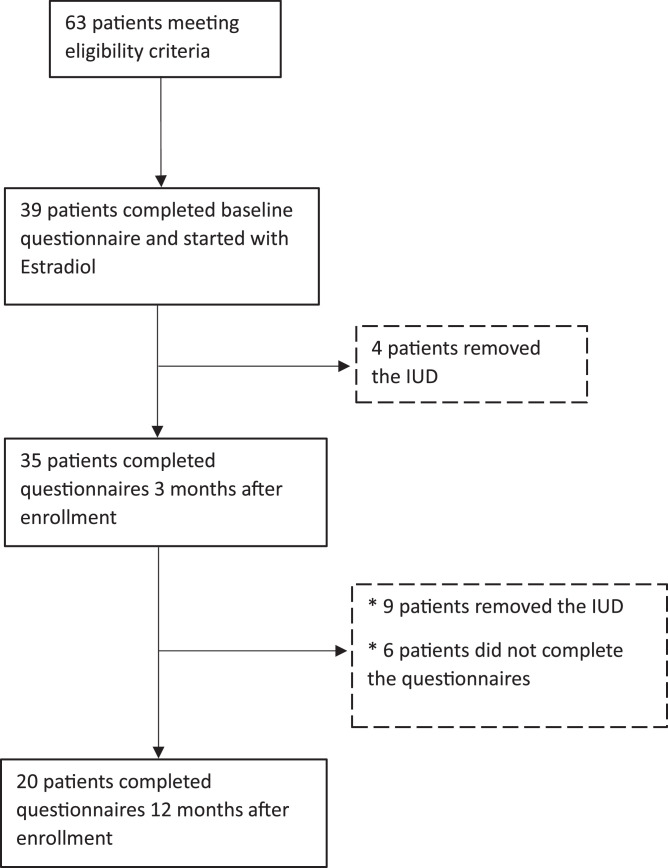
Flowchart of enrollment *IUD*, intrauterine device.

A total of 63 patients were eligible for this study, of whom 39 patients provided informed consent and completed the baseline questionnaires before starting estradiol treatment (Figure 1). The 52-mg LNG-IUD was removed in 4 patients within 3 months after the start of estradiol, leaving 35 patients for analysis at the 3 months of follow-up. At 12 months of follow-up, the 52-mg LNG-IUD was removed in 9 additional patients, and 6 patients were lost to follow-up, leaving 20 patients for the 12-month analyses. The baseline characteristics are presented in Table 1. The median age was 30 years (IQR, 23–37), and the median BMI was 23.0 kg/m^2^ (IQR, 22.0–26.5). The 52-mg LNG-IUD was inserted in 48.7% of patients for contraception, in 28.2% of patients for treatment of HMB, in 20.5% of patients for both contraception and HMB, and in 2.6% of patients for the reduction of symptoms caused by endometriosis. The median endometrial thickness on transvaginal ultrasound before insertion of the 52-mg LNG-IUD was 3.0 mm (IQR, 2.0–3.0). If patients had received previous treatments, these treatments are presented in Table 1.

Heijden. Treatment of bleeding in long-term 52 mg levonorgestrel intrauterine devices users. Am J Obstet Gynecol Glob Rep 2025.

**Table 1:** Baseline characteristics

	N = 39
Characteristics	Median (IQR) or n (%)
Maternal age, y	30.0 (23.0–37.0)
Body mass index, kg/m^2^	23.0 (22.0–26.5)
Parity	
Nulliparity	10.0 (52.6)
Multiparity	9.0 (47.4)
Indication of 52-mg LNG-IUD	
Contraception	19.0 (48.7)
Heavy menstrual bleeding	11.0 (28.2)
Contraception and heavy menstrual bleeding	8.0 (20.5)
Endometriosis	1.0 (2.6)
Number of previous LNG-IUSs	
0	27.0 (69.2)
1	6.0 (15.3)
2	4.0 (10.3)
4	1.0 (2.6)
Missing	1.0 (2.6)
Previous treatment	
No	26.0 (66.7)
Yes, lynestrenol	2.0 (5.1)
Yes, oral contraceptives	6.0 (15.4)
Yes, cryotherapy cervix	2.0 (5.1)
Yes, new LNG-IUD	1.0 (2.6)
Missing	2.0 (5.1)
Endometrial thickness, mm	3.0 (2.0–3.0)
Missing^a^	16.0
Insertion performed	
General practitioner	4.0 (10.3)
Gynecologist/resident	35.0 (89.7)

*LNG-IUD*, levonorgestrel intrauterine device; *LNG-IUS*, levonorgestrel intrauterine system.

a XXX.

### Primary outcome

**Figure 2:**
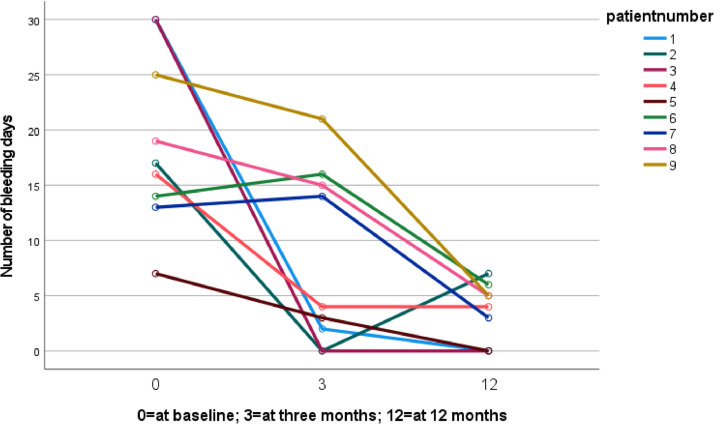
The course of bleeding days per patient at baseline, at 3 months, and at 12 months after use of estradiol A total of 9 patients had filled out the questionnaires at baseline, at 3 months, and at 12 months.

The mean number of bleeding days decreased significantly from 22.5 days per month at baseline to 12.8 days per month at 3 months after the start of estradiol treatment, as presented in Table 2. Figure 2 shows the changes in the number of bleeding days for 9 patients who had their 52-mg LNG-IUD in place throughout the entire follow-up period and completed all 3 sequential questionnaires regarding their number of bleeding days. Patients were not included if 1 or more questionnaires were missing, leaving only 9 patients to be included.

**Table 2:** Primary and secondary outcomes

Outcome	Baseline	At 3 mo	At 12 mo
Follow-up, n (%)			
Lost to follow-up	0 (0)	0 (0)	6 (15.4)
52-mg LNG-IUD removed^a^	0 (0)	4 (10.3)	13 (33.3)
52-mg LNG-IUD in situ^b^	39 (100.0)	35 (89.7)	20 (51.3)
Bleeding days, mean (SD)	22.5 (7.1)	12.8 (10.4)	3.5 (2.6)
95% CI for change	Reference	5.3–14.1	
*P* value for change	—	<.01^c^	Not calculated due to many missing data
Missing	3	3	10
Acceptable bleeding patterns, n (%)			
Yes	0 (0)	11 (34.4)	7 (50.0)
No	39 (100.0)	21 (65.6)	7 (50.0)
*P* value for change	Reference	<.03^d^	Not calculated due to many missing data
Missing	0	3	6
Satisfaction 52-mg LNG-IUD			
Yes	6 (16.2)	19 (65.5)	16 (94.1)
No	31 (83.8)	10 (34.5)	1 (5.9)
*P* value for change	Reference	<.01^d^	Not calculated due to many missing data
Missing	2	6	9
Excluded while the IUD was no longer in situ	0	4	13
Reason for 52-mg LNG-IUD removal (n=13)^a^	Not applicable		
Unacceptable bleeding		3	7
Side effects of estradiol		1	0
Side effect of LNG-IUD		0	1
Missing		0	1
Side effects of estradiol,^e^ n (%)	Not applicable^f^		Not applicable^f^
None		18 (51.4)	
Painful swollen breasts		10 (28.6)	
Mood swings		4 (11.4)	
Edema and/or weight gain		5 (14.3)	
Gastrointestinal complaints		3 (8.6)	
Fatigue		2 (5.7)	
Headache		3 (8.6)	
Vaginal discharge		21 (60.0)	
Missing while the IUD was no longer in situ		4	

*IUD*, intrauterine device; *LNG-IUD*, levonorgestrel intrauterine device.

a Cumulative number

b Included into the analysis

c Compared with baseline using the paired *t* test for paired observations

d Compared with baseline using the McNemar test

e Patients reported multiple side effects

f Patients were not using estradiol at this point in the study.

Heijden. Treatment of bleeding in long-term 52 mg levonorgestrel intrauterine devices users. Am J Obstet Gynecol Glob Rep 2025.

### Secondary outcomes

**Figure 3:**
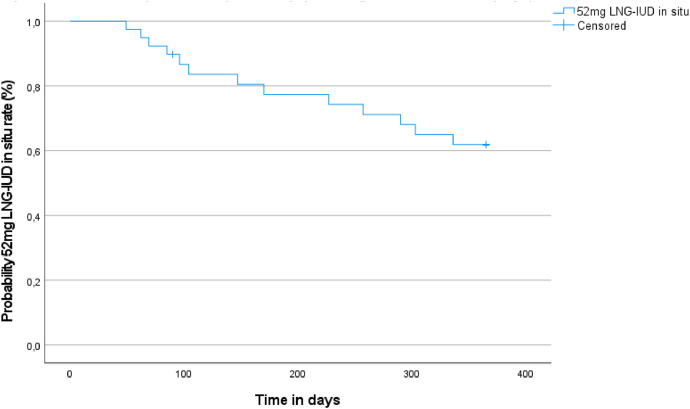
Kaplan-Meier survival plot of time (in 365 days) to 52-mg LNG-IUD removal (days) after treatment with estradiol For 6 patients, data were missing for removal at 12 months. At 3 months, all 6 patients had their 52-mg LNG-IUD in situ. Data were imputed as removal at 90 days (ie, after 3 months of follow-up). *LNG-IUD*, levonorgestrel intrauterine device.

The 52-mg LNG-IUD was removed in 10.3% of patients at 3 months of follow-up and in 33.3% of the patients at 12 months of follow-up, as shown in Table 2. Subsequently, at 12 months, 51.3% of patients still had the LNG-IUD in situ, whereas 33.3% of patients had it removed. However, data were missing for the remaining 15.4% of patients. The number of patients reporting acceptable bleeding patterns and satisfaction with the 52-mg LNG-IUD increased substantially over 12 months of follow-up (Table 2). The most frequent reason for the removal of the 52-mg LNG-IUD as reported by the patients was unacceptable bleeding pattern. At 3 months of follow-up, 1 patient removed the 52-mg LNG-IUD because of the side effects of estradiol treatment. The Kaplan-Meier survival curve of time (in days; ie, 12 months) to 52-mg LNG-IUD removal after estradiol treatment shows that 52-mg LNG-IUD removal occurred at a constant rate over the first year (Fig 3). Table 2 presents the reported side effects as reported during estradiol treatment.

Heijden. Treatment of bleeding in long-term 52 mg levonorgestrel intrauterine devices users. Am J Obstet Gynecol Glob Rep 2025.

## Discussion

### Principal findings

In this prospective observational study, a reduction in the number of bleeding days was observed 3 months after estradiol treatment in patients with irregular bleeding and a 52-mg LNG-IUD more than 6 months in place. Because of substantial loss to follow-up, conclusions regarding change in bleeding days 12 months after treatment could not be drawn. The 52-mg LNG-IUD was removed in 10.3% of patients after 3 months and in 33.3% of patients after 12 months. No adverse events were reported, but 20 patients reported side effects of estradiol treatment, including painful swollen breasts, headaches, mood swings, gastrointestinal complaints, fatigue, vaginal discharge, and weight changes.

### Results

The Dutch guideline for general practitioners recommends treatment with daily oral estradiol for 1 month for 52-mg LNG-IUD users with irregular bleeding patterns (https://www.nhg.org/standaarden/volledig/nhg-standaard-vaginaal-bloedverlies). The translation of this guideline is provided in the Supplementary text section. However, evidence supporting this treatment is lacking.

Here, the discontinuation rate of the 52-mg LNG-IUD was 33.3% (12 months after the start of the 6-week estradiol treatment). We observed that approximately 51.0% of patients did not remove the 52-mg LNG-IUD within 1 year (15.0% missing data). This is important to consider when counseling patients about their options in case of bleeding complaints during long-term 52-mg LNG-IUD use. Our trial reported a high rate of side effects. However, there were no adverse events. Although this study was not powered for this, estradiol seems to be a safe treatment option for patients with a 52-mg LNG-IUD in situ for at least 6 months who have unacceptable bleeding. However, women should be informed about possible side effects during treatment.

### Clinical implications

We hypothesized that estradiol will regenerate the endometrium during the use of the 52-mg LNG-IUD and decrease irregular bleeding. The results of our study support our hypothesis as we observed a significant reduction in reported bleeding days 3 months after the use of oral estradiol for 6 weeks.

### Research implications

Because of the design of the study, it is unclear whether bleeding patterns improved because of treatment with estradiol or whether bleeding patterns improved spontaneously. A randomized controlled trial (RCT) is proposed to further evaluate the effectiveness of estradiol as a treatment option for irregular bleeding in patients with a 52-mg LNG-IUD in situ for at least 6 months. To support the hypothesis and to further understand the pathophysiology behind bleeding disorders, it is recommended to assess the endometrium thickness during the follow-up period and take biopsies for histological evaluation of the endometrial and submucosal layers.

### Strengths and limitations

The current study has several limitations. First, the sample size of this prospective observational study is small (N = 39). There was no sample size calculated before the start of this study. This was not possible because of the lack of evidence on this matter. A larger sample size was aimed (100 patients), but the trial was stopped before it had reached its target number of patients because of the difficulty of enrolling patients due to the lack of eligible patients. The reasons for this increasing difficulty are unknown. There is a wide range of reasons for women to reject treatment with hormones. These include fear of negative experiences and side effects spread by social media.^10^ We have not registered how many patients declined treatment and why they did so.

Because of a high dropout rate, the 12-month bleeding results after the start of estradiol treatment have to be interpreted with caution. Bias may have occurred because of the selective dropout of nonresponders, leading to an overestimation of the reduction in bleeding days. Furthermore, we have no information as to whether patients received other treatment or had a reinsertion of an LNG-IUD for their bleeding complaints during the study period. As these treatments for unfavorable vaginal bleeding can affect outcome measurements, this is important to consider. It is important to note that more than 30% of patients removed their 52-mg LNG-IUDs before the 12-month follow-up. This likely influenced the final count of bleeding days, as the bleeding days of those who removed the 52-mg LNG-IUD were no longer included in the overall results. The single-arm observational design is a further shortcoming that limits the amount of evidence on the effect of estradiol treatment.

## Conclusions

In this prospective observational study, a reduction in the mean number of bleeding days was observed in long-term 52-mg LNG-IUD users with unfavorable bleeding after the administration of estradiol for 6 weeks. In addition, the satisfaction rates for bleeding patterns increased significantly. An RCT is proposed to further evaluate estradiol as a treatment option for unfavorable bleeding in 52-mg LNG-IUD users.

## CRediT authorship contribution statement

**Patty A.H.H. van der Heijden:** Writing – review & editing, Writing – original draft, Methodology, Investigation, Formal analysis, Conceptualization. **Karlijn A. Röttgering:** Writing – original draft, Formal analysis, Data curation. **Tamara J. Oderkerk:** Writing – original draft, Formal analysis, Data curation, Conceptualization. **Jeanne P. Dieleman:** Writing – review & editing, Validation, Supervision, Methodology, Formal analysis. **Arianne C. Lim:** Writing – review & editing, Supervision, Conceptualization. **Marlies Y. Bongers:** Writing – review & editing, Writing – original draft, Supervision, Methodology, Investigation, Formal analysis, Conceptualization. **Peggy M.A.J. Geomini:** Writing – review & editing, Writing – original draft, Supervision, Methodology, Formal analysis, Conceptualization.

## Appendix Supplementary materials

Image, application 1

## Footnotes

Supplementary material associated with this article can be found in the online version at doi:10.1016/j.xagr.2025.100474.
