# Supplementary material for: Treatment of unacceptable bleeding in long-term users of 52-mg levonorgestrel intrauterine device: a prospective observational study
Source: AJOG Glob Rep. 2025 Mar 20;5(2):100474. doi: 10.1016/j.xagr.2025.100474 (PMC12008142; doi:10.1016/j.xagr.2025.100474)
Supplement: Supplementary file 1 [file mmc1.docx]

**Supplementary text**

*Policy with progestogen-only contraceptives (hormonal IUD, etonogestrel implant)*

Little is known about the effectiveness of drug treatment of irregular bleeding in continuous use of progestogens (hormonal IUD or implantable rod). In a Cochrane review (33 RCTs, 3677 patients, search date 2012), the effectiveness of any treatment (such as oestrogen, NSAIDs, or tranexamic acid) was not demonstrated.

*Conclusion*

Based on experience, the guideline recommends trying estradiol 1 mg 1 dd for 1 month if there is much discomfort from blood loss, taking into account the contraindications to estrogen use.
